# Supplementary material for: A role for fibroblast and mural cell subsets in a nerve ligation model of neuropathic pain?
Source: Brain Behav Immun. Author manuscript; Available in PMC 2025 Nov 20. (PMC7618384; doi:10.1016/j.bbi.2025.05.012)
Supplement: Supplementary Material [file EMS210728-supplement-Supplementary_Material.zip › 1-s2.0-S0889159125001898-mmc2.zip!/Suppl_RNotebook_1a.rev.html]

scRNA\_seq of nerve mesenchyme


Code 

- Show All Code
- Hide All Code
- Download Rmd

# scRNA\_seq of nerve mesenchyme

Additional figures produced during revision period:


```
library(Seurat)
library(ggplot2)
library(tidyverse)
library(patchwork)
```


```
load("/Users/franziskadenk/Library/CloudStorage/OneDrive-SharedLibraries-King'sCollegeLondon/Denk Lab SharePoint - Mesenchymal_MS/scRNA_seq_data/nerve_mesenchymal_cells.Robj")
```


```
Idents(bmc) <- bmc$cluster_IDs
DimPlot(bmc, split.by="Injury", label = TRUE, cols = c("FB_Cldn1" = "#ffffba", "FB_Col15a1" = "#ffb3ba", "FB_Ccl19" = "#ffdfba", "FB_Pi16" = "#d5a6bd", "SC" = "#baffc9", "mySC" = "#f2f6c3", "MC" = "#bae1ff", "EC" = "#cccccc", "IC" = "#559e83"), pt.size=0.7)
```


```
Idents(bmc) <- bmc$seurat_clusters
DimPlot(bmc, split.by="Injury", label = TRUE, pt.size=0.7, cols = c("5" = "#ffffba", "2" = "#ffb3ba", "0" = "#ffdfba", "4" = "#d5a6bd", "1" = "#baffc9", "7" = "#559e83", "9" = "#f2f6c3", "8" = "#bae1ff", "3" = "blue","6" = "#cccccc", "10" = "#559e83"))
```


```
Idents(bmc) <- bmc$seurat_clusters
bmc <- (RenameIdents(bmc, `0` = "FB_Ccl19", `1` = "SC", `9` = "mySC", `8` = "MC", `2` = "FB_Col15a1", `6` = "EC", `5` = "Cldn1",`7` = "SC", `4` = "FB_Pi16", `3` = "MC", `10` = "IC"))
bmc[["cluster_IDs2"]] <- Idents(object = bmc)
```


```
Idents(bmc) <- bmc$seurat_clusters
plot1 <- DimPlot(bmc, split.by="Injury", label = TRUE, label.size = 5, cols = c("5" = "#ffffba", "2" = "#ffb3ba", "0" = "#ffdfba", "4" = "#d5a6bd", "1" = "#baffc9", "7" = "#559e83", "9" = "#f2f6c3", "8" = "#bae1ff", "3" = "blue","6" = "#cccccc", "10" = "#559e83"), pt.size=0.7) + theme(
  axis.text = element_text(size = 14),  
  axis.title = element_text(size = 16),  
  plot.title = element_text(size = 22), legend.position = "none")
Idents(bmc) <- bmc$cluster_IDs2
plot2 <- DimPlot(bmc, split.by="Injury", label = TRUE, label.size = 5, cols = c("Cldn1" = "#ffffba", "FB_Col15a1" = "#ffb3ba", "FB_Ccl19" = "#ffdfba", "FB_Pi16" = "#d5a6bd", "SC" = "#baffc9", "mySC" = "#f2f6c3", "MC" = "#bae1ff", "EC" = "#cccccc", "IC" = "#559e83"), pt.size=0.7) + theme(
  axis.text = element_text(size = 14),  
  axis.title = element_text(size = 16),  
  plot.title = element_text(size = 22), legend.position = "none")   

combined_plot <- (plot1 | plot2) + 
  plot_layout(ncol = 2, nrow = 1, widths = c(1.5, 1.5)) +
  plot_annotation(theme = theme(aspect.ratio = 0.5))

print(combined_plot)
```


```
library(cowplot)
custom_colours <- c("#DEEDCF", "#99D492","#56B870","#1D9A6C","#137177", "#0A2F51")
IL6 <- FeaturePlot(bmc, features = "Il6", min.cutoff = "q05", max.cutoff = "q95", order = TRUE, cols = custom_colours) + theme(legend.position = "none")
Ngf <- FeaturePlot(bmc, features = "Ngf", min.cutoff = "q05", max.cutoff = "q95", order = TRUE, cols = custom_colours) + theme(legend.position = "none")
Ccl2 <- FeaturePlot(bmc, features = "Ccl2", min.cutoff = "q05", max.cutoff = "q95", order = TRUE, cols = custom_colours) + theme(legend.position = "none")
Lif <- FeaturePlot(bmc, features = "Lif", min.cutoff = "q05", max.cutoff = "q95", order = TRUE, cols = custom_colours) + theme(legend.position = "none")
Notch3 <- FeaturePlot(bmc, features = "Notch3", min.cutoff = "q05", max.cutoff = "q95", order = TRUE, cols = custom_colours)+ theme(legend.position = "none")
Pdgfrb <- FeaturePlot(bmc, features = "Pdgfrb", min.cutoff = "q05", max.cutoff = "q95", order = TRUE, cols = custom_colours)+ theme(legend.position = "none")
Mcam <- FeaturePlot(bmc, features = "Mcam", min.cutoff = "q05", max.cutoff = "q95", order = TRUE, cols = custom_colours)+ theme(legend.position = "none")
Pdgfra <- FeaturePlot(bmc, features = "Pdgfra", min.cutoff = "q05", max.cutoff = "q95", order = TRUE, cols = custom_colours)+ theme(legend.position = "none")

combined_plot <- cowplot::plot_grid(
  Pdgfra, Pdgfrb, Mcam, Notch3,
  IL6, Lif, Ngf, Ccl2,
  ncol = 4, rel_widths = c(1.5, 1.5, 1.5, 1.5)  
)


print(combined_plot)
```


```
data <- data.frame(
  Gene = rep(c("Il6", "Ngf", "Lif", "Ccl2"), each = 12),
  Sample = rep(c("Kalinski", "Carr", "Wolbert", "Villa"), each = 3, times = 4),
  CellType = rep(c("Mural cells", "Pdgfrb+ fibroblasts", "Pdgfrb negative cells"), times = 16),
  Value = c(
    # Il6 data
    10.7, 3.4, 1.775, 8.4, 5.433333333, 4.266667, 1.1, 2, 0.316667, 5.7, 2.08, 3.7,
    # Ngf data
    28.4, 14.7, 0.5, 9.8, 3.166666667, 0.4, 3.3, 2.7, 0.066667, 16.2, 2.56, 0.233333,
    # Lif data
    19.5, 55.1, 2.35, 13, 29.06666667, 1.6, 2.2, 0.8, 0.083333, 4, 5.36, 0.266667,
    # Ccl2 data
    0.6, 11.5, 21, 0.2, 3.733333333, 11.83333, 0, 15.8, 5.216667, 0.4, 8.58, 2.166667
  )
)

data$Gene <- factor(data$Gene, levels = c("Il6", "Ngf", "Lif", "Ccl2"))
data$CellType <- factor(data$CellType, levels = c("Mural cells", "Pdgfrb+ fibroblasts", "Pdgfrb negative cells"))

data <- data %>%
  group_by(Gene, Sample) %>%
  mutate(Percent = Value / sum(Value) * 100) %>%
  ungroup()
```


```
head(data)
```


```
ggplot(data, aes(x = Gene, y = Value, fill = CellType)) +
  geom_bar(stat = "identity", position = "stack") +
  scale_fill_manual(values = c("Mural cells" = "darkgreen", "Pdgfrb+ fibroblasts" = "lightgreen", "Pdgfrb negative cells" = "grey")) +
  facet_wrap(~ Sample, ncol = 4) +
  labs(x = "Gene", y = "Expression", fill = "Cell Type") +
  theme_minimal() +
  theme(axis.text.x = element_text(angle = 45, hjust = 1))
```


```
# Ensure that Sample is a factor with the correct order
data$Sample <- factor(data$Sample, levels = c("Kalinski", "Carr", "Wolbert", "Villa"))

ggplot(data, aes(x = interaction(Sample, Gene, lex.order = TRUE), y = Percent, fill = CellType)) +
  geom_bar(stat = "identity", position = "stack", width = 0.8) + # Adjust bar width for spacing
  scale_fill_manual(values = c("Mural cells" = "darkgreen", "Pdgfrb+ fibroblasts" = "lightgreen", "Pdgfrb negative cells" = "grey")) +
  
  # Add facets for gene names above the groups
  facet_wrap(~Gene, scales = "free_x", strip.position = "top", ncol = 4) +
  
  # Override x-axis labels to show only sample names
  scale_x_discrete(labels = rep(c("Kalinski", "Carr", "Wolbert", "Villa"), times = 4)) +
  
  # Adjust theme
  theme_minimal() +
  theme(
    axis.text.x = element_text(size = 18, family = "Arial", angle = 45, hjust = 1), # Restore x-axis tick labels
    axis.text.y = element_text(size = 18, family = "Arial"), # Y-axis tick labels
    axis.title.y = element_text(size = 18, family = "Arial"), # Y-axis title
    legend.text = element_text(size = 22, family = "Arial"), # Legend text
    legend.title = element_text(size = 22, family = "Arial"), # Legend title
    strip.text = element_text(size = 22, family = "Arial", face = "bold"), # Gene names above bars
    panel.grid.major.x = element_blank(), # Remove vertical grid lines
    panel.spacing.x = unit(1, "lines"), # Add spacing between groups
    axis.title.x = element_blank() # Remove x-axis title
  ) +
  
  labs(y = "% Gene Expression", fill = "Cell Type")
```
